# A Study of the Peculiarities of the Formation of a Hybrid Interface Based on Polydopamine between Dental Tissues and Dental Composites, Using IR and Raman Microspectroscopy, at the Submicron Level

**DOI:** 10.3390/ijms241411636

**Published:** 2023-07-19

**Authors:** Pavel Seredin, Dmitry Goloshchapov, Nikita Buylov, Vladimir Kashkarov, Khidmet Shikhaliev, Andrey Potapov, Yuri Ippolitov, Viktor Kartsev, Sergey Kuyumchyan, Raul de Oliveira Freitas

**Affiliations:** 1 Solid State Physics and Nanostructures Department, Voronezh State University, University Sq. 1, 394018 Voronezh, Russiakashkarov@phys.vsu.ru (V.K.); 2 Laboratory of Organic Additives for the Processes of Chemical and Electrochemical Deposition of Metals and Alloys Used in the Electronics Industry, Voronezh State University, University Sq. 1, 394018 Voronezh, Russia; shikh1961@yandex.ru (K.S.);; 3 Department of Pediatric Dentistry with Orthodontia, Voronezh State Medical University, Studentcheskaya St. 11, 394006 Voronezh, Russia; 4 InterBioscreen, P.O. Box 218, 119019 Moscow, Russia; 5 Saint Petersburg State University Hospital, 154, Fontanka River Embankment, 198103 St. Petersburg, Russia; 6 Brazilian Synchrotron Light Laboratory (LNLS), Brazilian Center for Research in Energy and Materials (CNPEM), Campinas 13083-970, Sao Paulo, Brazil; raul.freitas@lnls.br

**Keywords:** polydopamine, dental materials, hybrid interface, dental tissue, biomimetic

## Abstract

The creation of buffer (hybrid) layers that provide improved adhesion to two heterogeneous materials is a promising and high-priority research area in the field of dental materials science. In our work, using FTIR and Raman microspectroscopy at the submicron level in a system of dental composites/intact dental enamel, we assessed the molecular features of formation and chemically visualized the hybrid interface formed on the basis of a nature-like adhesive, polydopamine (PDA). It is shown that a homogeneous bioinspired PDA–hybrid interface with an increased content of O-Ca-O bonds can be created using traditional methods of dental tissue pretreatment (diamond micro drilling, acid etching), as well as the subsequent alkalinization procedure and the developed synthesis technology. The development of the proposed technology for accelerated deposition of PDA–hybrid layers, as well as the creation of self-assembled biomimetic nanocomposites with antibacterial properties, may in the future find clinical application for minimally invasive dental restoration procedures.

## 1. Introduction

A modern biomimetic strategy for dental hard tissue restoration requires the use of materials that meet the following requirements in terms of their physico-chemical and morphological characteristics, as well as their hierarchical structure [1,2,3,4]. Such materials should possess the maximum affinity to the natural dental tissue in phase and molecular composition, and at the same time be able to form a stable bond with the organomineral matrix of the teeth at the interface [1,5]. As a result of the biomimetic therapeutic approach it is possible to achieve not only maximum preservation of the intact enamel/dentin layer, but also to increase the lifetime of the restored area, as well as to ensure greater preservation of the tooth as a whole [3,4]. Therefore, a trend in modern dental material science is the development of a new class of hybrid biomaterials for hard tissue restoration with the above-mentioned properties [6,7,8]. To date, attempts have already been made to “regenerate” dental hard tissues, such as enamel, using synthesized mineral agents or specialized substances related to biogenic hard tissues [1,3,9,10]. However, the significant difference in the functional properties of modern restorative materials with respect to those of natural enamel/dentin, as well as the low chemical tropism of biocomposites to natural dental tissue, is a significant problem. Therefore, the durability of restorations is still an open question [1,11].

It has been repeatedly shown that the quality and durability of a completed dental restoration is determined by the stability of the formed biocomposite/dental tissue bond [12], as well as the state of the interface, which in turn depends on its pretreatment technology [4,13,14]. Therefore, in order to increase the level of bonding of dissimilar materials in dental restorations, it is necessary to maximize the bonding of the materials used [5,15]. In modern commercial bonding systems, cohesion occurs through the formation of a hybrid transition layer and adhesion of the adhesive components to the array of micropores formed as a result of etching of the natural tissue [12,16]. However, commonly available commercial restorative systems, for all their versatility and convenience, still do not provide sufficient and durable adhesion to enamel [11,17].

In our opinion, the optimal strategy for dental tissue restoration within the framework of the mentioned biomimetic approach is the formation of a hybrid bioinspired layer that can simultaneously bind actively not only to the morphological basis of dental tissue (calcium hydroxyapatite and enamel matrix proteins, which play a vital and fundamental role in enamel functioning), but also to the restorative material. As a result, improved integration of the synthetic systems with dental hard tissues can be achieved.

One such approach is the use of a nature-like adhesive, polydopamine (PDA), whose films are perfectly formed at interphase interfaces of different types (solid–liquid, liquid–liquid, water–air) [18,19,20]. PDA has been repeatedly shown to be an interesting and promising biomimetic material for the synthesis of multilayer films, stable bioinspired encapsulated nanoparticle nanocomposites, PDA-decorated proteins, etc. [18]. Due to the large number of heterogeneous chemical bonds and the flat structure of the molecule, polydopamine forms strong bonds with the surface of almost all known materials during polymerization [21,22]. The high adhesion values of PDA and the exceptional mechanical properties of the composites formed on its basis mean there are a wide range of applications of such a bioinspired adhesive. Among them, dental applications (technologies) are among the most in-demand ones [23,24].

The main problem arising at the stage of construction of bioinspired nanocomposites and related to the layered structure of PDA is the need for chemical visualization and differentiation of the organic-mineral molecular groups included in the latter at the submicron level, as well as the analysis of the emerging chemical bonds between the components. For this purpose, it is advisable to use IR and Raman imaging techniques and subsequent multispectral analysis, which allow the classification of the identified features using molecular spectroscopy signatures [25,26,27,28,29,30]. Moreover, the combination of microscopy using high-numerical-aperture optics with an FTIR spectrometer allows an optimal signal-to-noise ratio for point spectra to be achieved. At the same time, the FTIR reflection mode useful for studies of molecular changes in the region of interest (chemical imaging) with high spatial and spectral resolution [25,26,31].

It should be noted that many works are devoted to the peculiarities of the formation of thin PDA films. At the same time, in our opinion, insufficient attention has been paid to the issue of creating a PDA dental hybrid interface, the interaction between polydopamine and natural hard dental tissue, and the functionalization of the bond formed at the stage of restoration. The available data are illustrative rather than exhaustive, and require additional research. 

Therefore, the aim of our work was to investigate the formation of a polydopamine-based hybrid interface between dental enamel and dental composites using high-resolution FTIR and Raman microspectroscopy at the submicron level.

## 2. Results and Discussion

The conditions for the polymerization of dopamine play a crucial role in the rate of deposition of PDA coatings [22,32,33]. Often, the speeding up of the polymerization process results in the formation of large agglomerates and PDA globules on the surface of a substrate [32]. Therefore, in order to provide an exaggerated formation of the uniform PDA coatings, we employed a technique proposed in [34]. To confirm that the employed technology proved to be a reliable approach to the fast creation of PDA coatings with sufficient thickness and uniformity on the surface of different materials (including dental tissue) under the same conditions (see Section 3.1), PDA was deposited on the silicon plate. Such an approach allowed us to control not only the quality of deposition, but also the roughness and uniformity of the deposited PDA films, since unlike the silicon plate, the surface of dental enamel has its own well-developed micro-morphology, which is formed due to the pre-treatment procedures.

**Figure 1:**
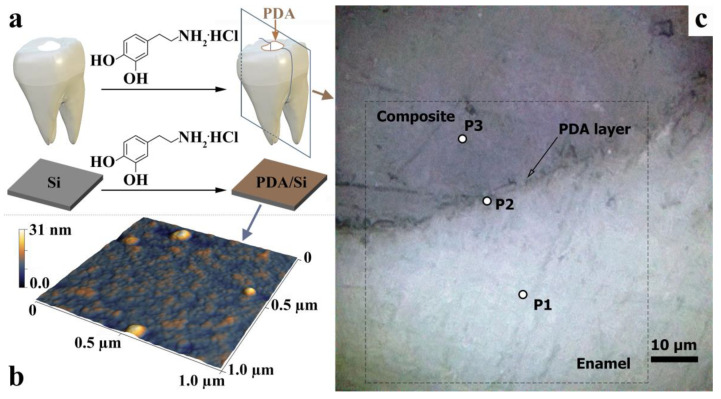
(**a**) AFM images of the PDA-coated silicon wafer. (**b**) AFM image of micro-region of PDA film deposited on the silicon plate. (**c**) Optical image of the hybrid interface at ×600 magnification. P1, P2, and P3 are areas from which Raman spectra were obtained. The dotted line indicates the 60 × 60 μm^2^ area wherein the IR mapping was performed.

Figure 1b represents an AFM image of thet micro-area on the surface of a PDA film deposited on the silicon plate. The results of AFM demonstrate that the PDA film is formed by the uniform distribution of a set of nanoparticles. The surface of the obtained film is characterized by a roughness of ~2.5 nm and a mean-square roughness is of ~4 nm, while the difference in height is ~30 nm; this correlates well with the results of [34]. This work was used as the basis for the elaboration of technology for exaggerated PDA deposition. At the same time, spontaneous agglomeration of nanoparticles can be seen in the AFM image (see Figure 1b), which may be due to the double deposition of the film on the substrate surface. The data obtained in our work confirm the idea that in the case of the technique employed for the accelerated deposition of the film, the thickness of the PDA coating increases linearly with the time of deposition [34]. Under the chosen technological conditions used in our work, the thickness of the PDA film was about 300 nm.

A typical light-field optical image of the denture/dental composite interface area is shown in Figure 1c. The area of the dental composite, the hybrid layer, and the natural enamel are clearly visible in contrast. It should be noted that the optical images of the interface show macro-relief, namely micron inhomogeneities in the area of the interface (Figure 1c). The formation of this macro-relief is due to the nature of cavity preparation in the dental tissue using a microturbine tip with a diamond bur, as well as the subsequent acid etching and leaching of the cavity walls (see preparation technique). The visualized micro-relief is typical of the selected preparation conditions [35,36].

It should be noted, however, that the optical images clearly do not show a formed PDA hybrid layer, which is due to both the homogeneous deposition of the PDA film on the surface of the preconditioned cavity and its thickness.

**Figure 2:**
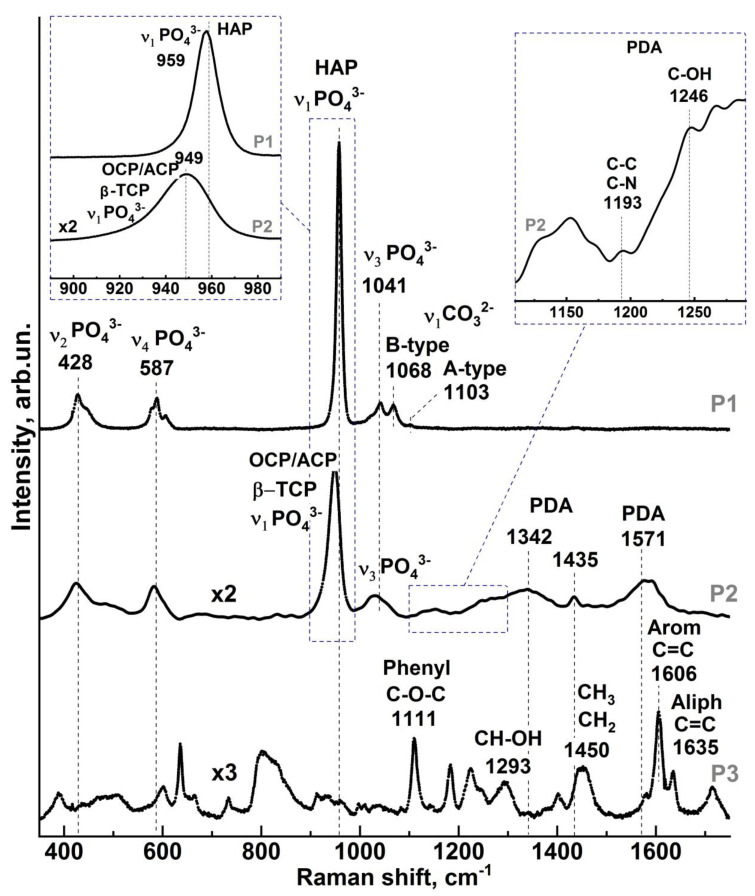
Raman spectra in different areas of the PDA hybrid interface: P1—enamel area near the interface, P2—PDA hybrid interface area, P3—dental composite area.

Raman spectra were collected to clarify the local phase and molecular composition at various points in the vicinity of the interface (see Figure 2). The spectra were obtained from the following areas: P1—enamel, P2—hybrid interface, and P3—dental composite. Note that using Raman microspectroscopy, the signal was collected from an area of ~1 μ^2^. At least ten single-type spectra were obtained in each of the three zones for all samples studied. The Raman modes in the spectra of the same-type areas did not differ in position and had slight differences in intensity and width at half of the band (FWHM). Therefore, the sample-averaged Raman spectra from the three interface regions are shown in Figure 2.

Analysis of the experimental Raman spectra from the region of healthy enamel (Figure 1, point P1) showed that the main intense maxima correspond to vibrations of phosphate and carbonate groups in the carbonate-substituted calcium hydroxyapatite (Figure 2, P1 spectrum), which is the basis of the mineral component of enamel [37,38,39,40,41,42]. The maxima attributed to phosphates are associated with four groups of υ_1_ to υ_4_ bands in the spectrum (Figure 2 P1 spectrum). The most intense of these, υ_1_ PO_4_^3−^, is located in the region of 930–990 cm^−1^ [37,38,40,41,42,43]. In contrast to carbonate-substituted hydroxyapatite [42,44], this oscillation in the spectrum of healthy natural enamel is shifted to the low-frequency region and localized around 959 cm^−1^ (see inset to Figure 2), which correlates with the known works [42,45,46,47,48,49,50]. It should be noted that an accurate position of this mode in the spectrum depends on orientation of hydroxyapatite crystals relative to the direction of the primary laser irradiation and its polarization [42,48]. In a number of works, it was shown that PO_4_^3−^ symmetric stretching mode can be localized in the range of 958 cm^−1^ [38], 959 cm^−1^ [42,45,48,49,50], and 960 cm^−1^ [39,51,52], while in case of fluorosis, it is located at about 962 cm^−1^ [53,54]. In our work, we investigated the slices of dental enamel cut in parallel to the occlusion surface of the tooth crown from its middle part (see section Design of the study, Figure 1a). Taking into account the use of micro-Raman spectroscopy technique and bearing in mind that scanning was performed orthogonally to the planes of enamel slices, the position of the main maximum of PO_4_^3−^ in this work was ~959 cm^−1^, which thus corresponds to the known data [42,45,46,47,48,49,50]. 

The υ_3_ PO_4_^3−^ band in the Raman spectra of healthy enamel consists of several overlapping modes localized in the range 1020 cm^−1^ to 1045 cm^−1^. It should be noted that the position and relative intensities of the υ_3_ PO_4_^3−^ band components are typical of the spectrum of natural enamel apatite [42,45]. A similar pattern can be observed for the υ_2_ and υ_4_ PO_4_^3−^ bands. These bands are formed by two and four overlapping peaks in the region of ∼420 cm^−1^ to 440 cm^−1^ and ~575 cm^−1^ to 615 cm^−1^, respectively, which agrees with the available literature data [37,38,40,41,42,43].

The presence of carbonates in the composition of healthy enamel, and in particular the inclusion of CO_3_^2−^ in the crystal lattice of apatite, is reflected in Raman spectra as an active vibrational mode υ_1_ CO_3_^2−^. It should be noted that in various calcium apatites, two variants of carbonate anion substitution of functional groups in lattice are possible. In B-type carbonated apatites, CO_3_^2−^ ion substitutes phosphate PO_4_^3−^ (typical for biogenic apatites [35,37,39,41,55,56]), which leads to the appearance of Raman spectra with maxima in the 1068–1071 cm^−1^ range. It should be also noted that the intensity of this mode varies in its dependence on the local features of dental tissue [39,55], just as the PO_4_^3−^ symmetric stretching mode depends on the orientation of the apatite nanocrystals in tooth enamel [42]. Therefore, the CO_3_^2−^ mode in the range of 1068 cm^−1^ is overlapped with the υ_3_ PO_4_^3−^ band localized near 1075 cm^−1^. In second variant (A-type substitution) incorporation of carbonate anion CO_3_^2−^ in the lattice of apatite occurs instead of the OH group that is typical of natural enamel [37,39,40], and leads to the appearance of a wide band in the 1010–1100 cm^−1^ region of the Raman spectrum. Analysis of this region in the intact P1 enamel spectrum demonstrates the presence of a low-intensity vibration with a maximum near 1103 cm^−1^, meaning a low content of A-type substitution, and at the same time, a more intensive CO_3_^2−^ mode in the range of 1068 cm^−1^ indicates at a predominant B-type carbonate substitution in the enamel apatite [37,38,40,41,42,43]. 

Examination of the spectrum from the hybrid interface region (see Figure 1c, point P2) and comparison with the P1 spectrum from the healthy enamel region revealed several features. These are related both to the rearrangement of the intensities of the maxima associated with the phosphate groups, and to the appearance of oscillations in the 1200–1650 cm^−1^ range in the P2 spectrum. Thus, in the spectrum of P2 from the region of the hybrid interface, as well as in the spectrum of natural P1 enamel (Figure 2), the most intense υ_1_–υ_4_ vibrations of phosphate groups are present. However, as can be seen from the inset to Figure 2 in the spectrum of P2 for υ_1_ PO_4_^3−^ mode, a significant (~10 cm^−1^) low-frequency shift is fixed in relation to its position in the spectrum of healthy enamel P1, as well as increasing FWHM. The change of the band profile in the region of 930–990 cm^−1^ in the Raman spectrum of P2 is due to the changes in the phase composition in the near-surface area, which take place as a result of enamel etching in 38% phosphoric acid and treatment in 0.016% solution of calcium hydroxide. The most probable calcium orthophosphates, which appear as a result of the performed pretreatment, prove to be the phases of amorphous calcium phosphate (ACP), dicalcium phosphate dihydrate (DCPD), octocalcium phosphate (OCP), and β-tricalcium phosphate (β-TCP) [57,58]. In non-equilibrium conditions, these phases can appear in mineralized tissue in different ratios. Therefore, at low concentration of these phases, their vibrational bands are overlapped, as in Raman spectra [57]. So, the observed asymmetrical spectral band in the range of 930–990 cm^−1^ is a result of the overlapping of bands characteristic of each of the assumed phases; the appearance of additional active maxima around 954 cm^−1^ is associated with ACP and OCP [57], around 949 cm^−1^ and 970 cm^−1^ is associated with β-tricalcium phosphate (β-TCP) [58,59], and around 986 cm^−1^ is associated with dicalcium phosphate dihydrate (DCPD) [57]. Similar changes (frequency shift and spectral profile change) are observed in the P2 spectrum for the maxima associated with PO_4_^3−^ phosphate groups and localized in the regions 1020–1045 cm^−1^, 420–440 cm^−1^, and ~575–615 cm^−1^.

In addition to the features noted, the Raman spectra from the P2 region contain additional broad vibrational bands localized in the range 1200–1650 cm^−1^, characteristic of carbonaceous materials whose frequencies and spectral profiles coincide with those usually observed in PDA-based materials [19,22,60].

In addition, C-H in-plane deformation near 1190 cm^−1^, C-C and C-N stretching in pyrrole, and C-OH stretching in the phenolic group near 1242 cm^−1^ (see inset to Figure 2), which are attributed to eumelanin and carbon materials having similar PDA-like supermolecular highly ordered superstructures, are observed at a low level, but are discernible [19,61]. 

This fact confirms the formation of a PDA layer on the surface of pre-treated enamel as a result of the synthesis procedure performed.

Analysis of the spectrum from the dental composite area confirms that in the P3 spectrum from the dental composite area in the range of 1200–1650 cm^−1^, no vibrations which are associated with PDA and are active in the P2 spectrum are observed. In the P3 Raman spectrum, the modes that can be attributed to the molecular groups of the materials included in the Synergy D6 flow dental material are active: 1110 cm^−1^ C-O-C (Phenyl), 1293 cm^−1^—CH-OH, 1430–1490 cm^−1^—CH_2_/CH_3_, 1600–1650 cm^−1^—aromatic and aliphatic C=C [62].

### 2.1. FTIR-Microspectroscopy

#### Chemical Mapping

**Figure 3:**
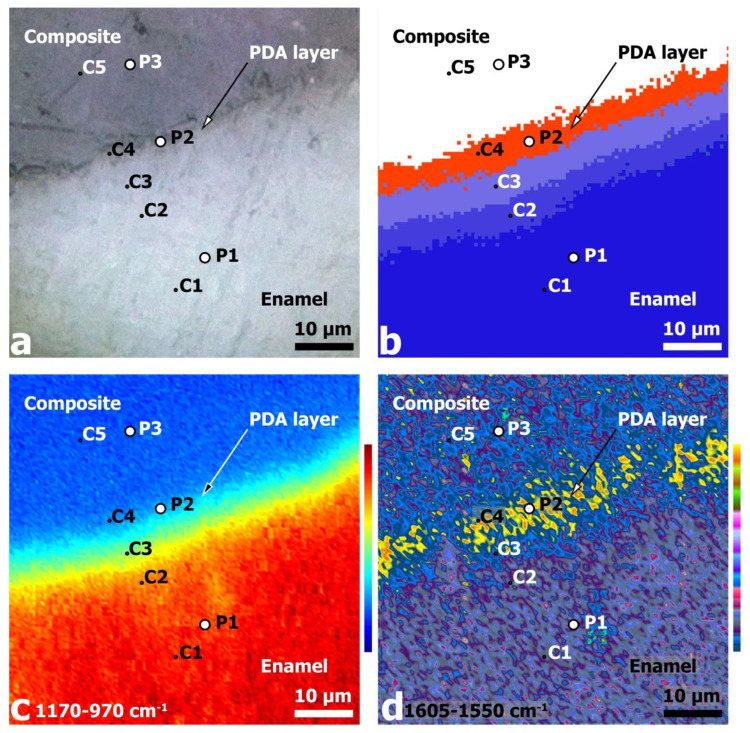
Images of the 60 × 60 μm^2^ region near the PDA hybrid interface: (**a**) optical image at ×600 magnification; (**b**) HCA analysis results; (**c**) IR absorbance (chemical) image of the surface area based on integration of 1170–970 cm^−1^ phosphate band intensity; (**d**) chemical image based on the integration of 1605–1550 cm^−1^ band intensity.

A 60 × 60 μm^2^ interface area was mapped for each of the samples obtained in this study using an IR microscope operating in reflection mode. An optical image of a typical mapped area of one of the samples is shown in Figure 3a. Despite the reflection mode, the micro-FTIR point spectra and hyperspectral maps are presented in absorbance units.

The hyperspectral data collected for all samples, such as FTIR maps, were analyzed using hierarchical cluster analysis (HCA). HCA allows for the grouping of an array of spectroscopic information (spectra) according to their spectral response. The classification results of the HCA are presented in the form of images, within which the points showing similar spectral responses (due to having minimal intra-cluster spectral differences) are coded by the same color. In this case, the maximum inter-cluster differences will be in regions with different spectral responses [63]. In contrast to optical microscopy, analytical HCA provides unambiguous insight into the shape and boundaries of regions with specific molecular composition [25,64].

The HCA of the FTIR hyperspectral dataset (see Figure 3b) reveals a clear segmentation of regions in the hybrid DPA interface area in the spectral region chosen for the analysis and the proximity function. The HCA results show that five different C1–C5 clusters are reliably detected in the analyzed region.

Comparison of the optical (Figure 3a) and HCA images (Figure 3b) of the analyzed area suggests that the first three clusters C1–C3 are located in the dental tissue (enamel) zone, cluster C4 is in the PDA zone of the hybrid layer, and cluster C5 covers the zone of the dental composite. It should be noted that the boundaries of the clusters have a shape similar to that of the pre-drilled diamond micro drill boundary of the dental tissue, i.e., there is a close to linear spatial delineation of the clusters.

For additional spectral visualization of the obtained hyperspectral dataset and comparative analysis, chemical imaging of the mapped areas from the interface zone was performed. The phosphate vibrational mode υ_3_ PO_4_^3−^ 1170–970 cm^−1^, which is associated with enamel apatite [53,64,65], and the most intense mode in the spectrum of polydopamine, localized around 1605–1550 cm^−1^ [60,66], were used for this purpose. The oscillation frequencies were chosen based on data from the literature, in which micro-FTIR reflectance chemical imaging has been used to investigate polydopamine-based dental hard tissues and layers [35,53,64,65,66]. Surface chemical images (see Figure 3c,d) were constructed by integrating the intensity of the indicated absorption bands using CytoSpec v. 2.00.07 software. The analysis of the chemical maps allowed us to unambiguously establish the distribution of phosphate (enamel) and polymer (PDA) phases in the analyzed area. Color bars were used to encode the integral intensity of the oscillations selected for chemometrics (see Figure 3c,d).

The chemical image constructed from the intensity distribution of the phosphate υ_3_ PO_4_^3−^ mode allows not only a visualization of the mineral component distribution, but also a clear definition of the phase boundary (interface) enamel/dental composite. The intensity of the phosphate maximum shows a progressive increase as one goes deeper into the enamel on the interface side, which is associated with an increase in phosphate content in the enamel apatite, as well as an increase in the crystallinity of the apatite. These changes are related to the pre-treatment of dental tissue and the presence of calcium orthophosphates phases (most probable ACP, OCP DCPD, and β-TCP) of non-stoichiometric compositions in the interface zone, which in turn correlates with Raman microspectroscopy data (see Figure 2). In addition, a mineral gradient can be seen in the phosphate chemistry image (Figure 3c) [65]; there are individual areas with a higher intensity, which is related to the morphology of natural enamel, i.e., the existence of enamel rods (enamel prisms) and interrod substance [67,68].

Regarding the chemical image of polydopamine from 1605–1550 cm^−1^ (Figure 3d), the highest integral intensity of this mode is localized in the region of the hybrid interface between the enamel and dental composite, which coincides with the spatial distribution of the C4 cluster in the HCA image (Figure 3b). It is important to note that the width of the polydopamine localization region is significantly greater than the expected PDA layer thickness for the selected synthesis conditions. On the other hand, analysis of the chemical map plotted against the band associated with the PDA shows that the maximum intensity of this phase is concentrated at different points in the hybrid interface. The results of spectral analysis and chemical mapping (see Figure 3), as well as consideration of the depth of analysis for FTIR reflection mode, indicate that the experimentally recorded DPA width of the hybrid layer is determined by several factors. Firstly, when considering the area of polydopamine localization, it can be seen that it coincides with the interface zone, the boundary of which has a characteristic microrelief with ~3 μ drops due to the cavity preparation in the enamel with a diamond micro drill (Figure 3a). Secondly, after the formation of the cavity in the dental tissue, the enamel walls were etched using a 38% phosphoric acid solution followed by treatment of the cavity walls with calcium hydroxide solution (see sample preparation technique). It has been repeatedly shown that etching with phosphoric acid results not only in the removal of the lubricated layer of dental tissue and functionalization of the enamel surface, but also in the formation of micro-relief associated with non-uniform etching of the enamel cores [5,69]. Therefore, the overall surface topography of the cavity walls in enamel after pre-treatment procedures has a high roughness value, where the difference between the lower and upper point can be ~3–4 μ. It should be taken into account that the polydopamine synthesis method used in this work allowed us to obtain relatively thin layers (~50–100 nm), which are deposited on the entire surface of the formed cavity. Taking into account the two-step process of polydopamine deposition implemented in our study, the thickness of the PDA film, according to the calculations performed, should not exceed 300 nm. This fact explains the presence of the polydopamine film at each section of the formed interface, regardless of the surface roughness value. It is noteworthy that the hybrid layer thickness determined from the HCA results (Figure 3b) coincides with that determined from the PDA chemical mapping (Figure 3d). This further confirms the distribution features of the film on the surface with the pre-prepared enamel micro-relief.

**Figure 4:**
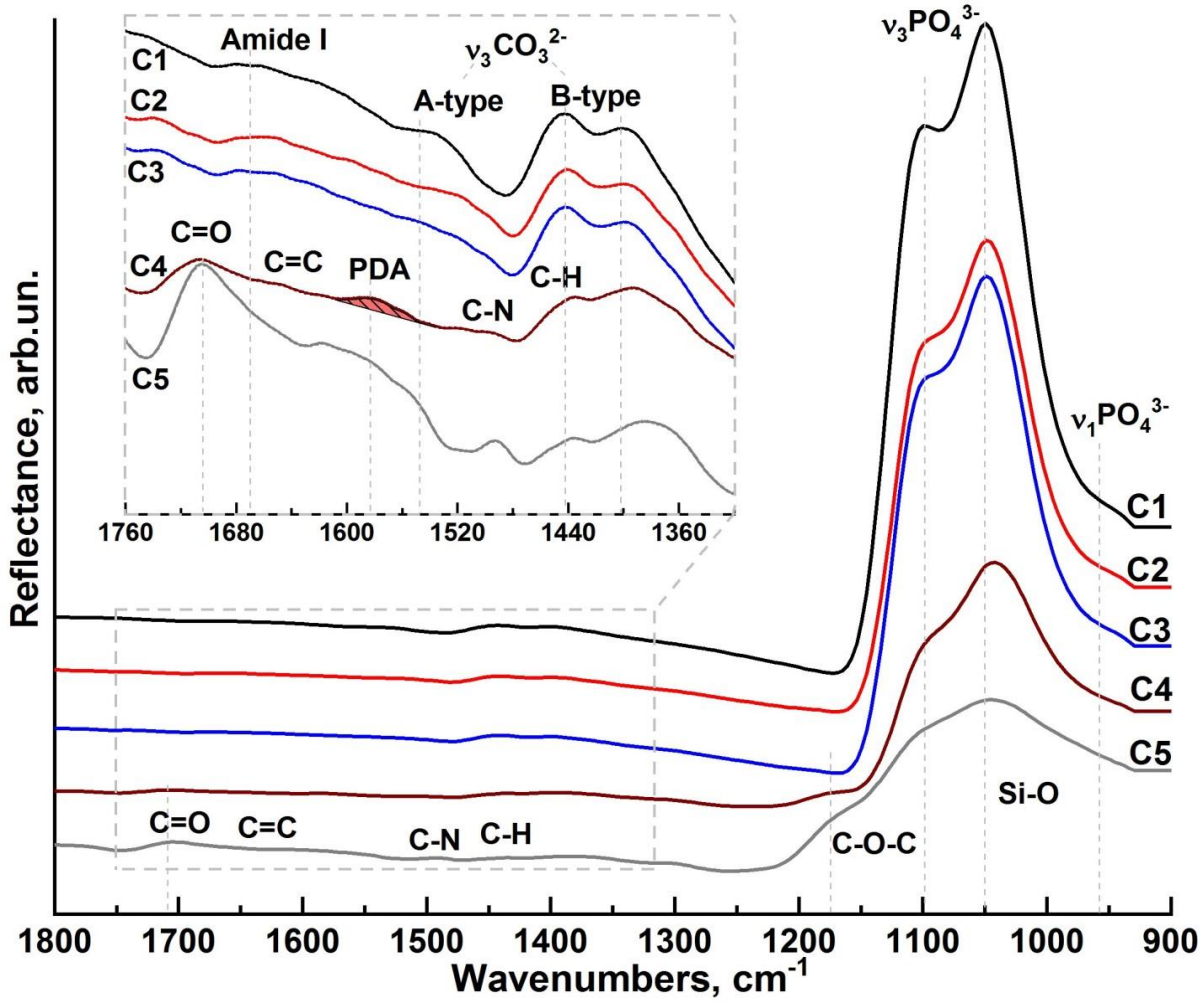
Averaged micro-FTIR reflectance spectra: C1—cluster spectrum from intact enamel; C2 and C3—cluster spectra from the area near the interface; C4—cluster spectrum coinciding with the hybrid interface area; C5—cluster spectrum from the area of the dental composite.

The analysis of averaged FTIR spectra allowed us to clarify the molecular composition characteristics of each cluster. For this purpose, at least 30 spectra were selected from typical zones (clusters C1–C5, Figure 3b HCA) for each of the samples. Then, standard spectral data processing procedures were performed for each set of spectra: smoothing, baseline corrections, normalization, and averaging. The averaged FTIR spectra obtained in this way for each cluster are presented in Figure 4. The analysis of the spectra is based on a set of known papers describing FTIR spectroscopy studies in the reflectance mode of dental hard tissue, dental composites, and PDA-based materials [35,70,71,72,73,74].

A comparative analysis of FTIR spectroscopy results revealed that the C1 cluster spectrum (Figure 4, C1 spectrum) has features characteristic of intact enamel [35,53]. The set of vibrational modes active in the FTIR spectrum is associated with both the mineral (calcium-phosphate and carbonate) and organic (tab to Figure 4, C1 spectrum) components of hard dental tissue.

The broad absorption band in the region of 1175–925 cm^−1^ in the C1 cluster spectrum represents the overlapping maxima of the triply degenerate asymmetric stretching mode, which is associated with the ν_3_ PO_4_^3−^ phosphate group vibrations [64]. Nearby, around 956 cm^−1^, a weak ν_1_ PO_4_^3−^ (symmetric stretching) band is detected in the infrared spectrum from the region of healthy enamel. The carbonate component of enamel is identified by the presence of characteristic maxima at 1545 cm^−1^, 1442 cm^−1^, and 1401 cm^−1^, which belong to the antisymmetric stretching band ν_3_ CO_3_^2−^ (inset to Figure 4, C1 spectrum) in the IR spectra. These vibrations occur when hydroxyl or phosphate radicals are replaced by CO_3_^2−^ groups (A and B-type substitutions, respectively). Active in the spectral range near 1545 cm^−1^ are the ν_3_ CO_3_^2−^ bands characteristic of A-type substitution, and in the range of 1450–1410 cm^−1^, the bands characteristic of B-type substitution. The simultaneous presence of ν_3_ CO_3_^2−^ vibrational modes correlated with A- and B-type substitution in the spectra indicates a mixed AB-type substitution of enamel apatite, which is in agreement with the known literature data [53,64,65]. Analysis of the low-intensity vibrational modes in the 1600−1500 cm^−1^ region (tab, Figure 4, C1 spectrum) and associated with the organic component shows that the maximum localized around 1650 cm^−1^ can be attributed to the C=O amide I valence vibrations of the protein component in enamel. The low intensity of the observed mode is explained by the low content of the organic component, which is 3–5% in intact enamel.

A detailed examination of the 1700–1400 cm^−1^ band (inlay, Figure 4, C2, C3 spectrum) reveals a decrease in the organic component as well as A-type replacement carbonates in C2 and C3 as compared to a healthy enamel zone C1, as indicated by the reduced intensity of the corresponding vibrational modes. With all this, the amide band overlaps the carbonate band of the A-type carbonated apatite; therefore, the decrease in intensity in this range is not only suggestive of a decrease in the A-type carbonated apatite, but could be related to the weakening of amide I. All this indicates that phase changes are occurring in the dental tissue when it is re-treated with a diamond micro drill and subsequent etching/alkalisation procedures. It should be noted that an even greater transformation of the spectral profile is observed for the C4 cluster. A noticeable frequency shift of the phosphate band ν_3_ PO_4_^3−^ is detected, as well as a change in the intensity of vibrations associated with the carbonate anion CO_3_^2−^. This result correlates with Raman spectroscopy data and indicates the presence of additional phases of ACP, OCP, DCPD, and β-TCP in the interface region. At the same time, no absorption bands associated with the organic component of enamel were observed in the C4 spectrum from the hybrid interface region. However, with that said, in the same spectrum, a broad band around 1585 cm^−1^ is present (tab to Figure 4, C4 spectrum), which is associated with the stretching modes ν_ring_(C=C) and ν_ring_(C=N) and indicates PDA formation [19], whose presence in this interface region was also detected using Raman microspectroscopy. A number of vibrations simultaneously active both in the C4 IR spectrum and in the reflectance spectrum from the C5 cluster can be ascribed to the C=O, C=C, C-N, and C-H groups as well as to the Si-O bonds included in the dental composite.

Crystalline quality as well as similarities and differences in crystal and molecular structure in different areas of enamel (C1-C3 clusters) and the PDA–hybrid interface (C4 cluster) can be estimated from the analysis of changes in the spectral profile of the phosphate vibration group. According to the available literature data, the deconvolution of the ν_3_ PO_4_^3−^ vibrational band profile in the 1175–925 cm^−1^ region allows us to identify the overlapping secondary phase vibrations. In this regard, it is important to note that the IR absorption spectra for various calcium phosphates have been widely described in the literature [56,74,75,76], including the differences in their vibrational spectra, with an indication of features in the position of absorption bands in the presence of defects [74]. At the same time, the rearrangement of the band correlated with the phosphate ion PO_4_ in the IR reflectance spectra of biogenic phosphates, similar to those obtained in our work, has been previously discussed, and its features, which are not observed or are less pronounced in the IR absorption spectra, have been described.

Therefore, for correct interpretation of the obtained spectral data, attention should be paid to the results of theoretical studies [77,78]. Thus, in [77], using a quantum chemical methodology, it was shown that in the IR spectrum of calcium-phosphate clusters, the maxima around 1092.7 cm^−1^ and 1073.6 cm^−1^ are the result of the overlap of the low-intensity ν(P-O) vibration and the average intensity of the δ(Ca-O-H) mode, while the bands around 1035.6 cm^−1^ and 1014.4 cm^−1^ refer to the ν(P-O), δ(O-P-O), and δ(Ca-O-P) bonds, respectively. The described features coincide with those observed in the reflection spectra in this work.

At the same time, a number of works devoted to theoretical and experimental studies of vibrational spectra of calcium phosphates, which are typical of natural enamel apatite, indicate that in different calcium phosphate nanostructures, depending on the stoichiometry and local atomic environment, vibrational frequencies can be observed, attributed to P-O bonds [56,74,75,76] Ca-O [79], phosphorus–oxygen bonds, and coordinated calcium, O-Ca-O, at about 1025 cm^−1^ [78], as well as uncoordinated P-O valence vibrations in the case of compact calcium phosphate about 1085 cm^−1^ [78]. In the works of Sandra Diez-García et al. and Babot-Marquillas et al., in which natural enamel was studied via FTIR reflection methods it is shown that the secondary phase vibrations in stoichiometric hydroxyapatite are Ca-O-P at 1103 cm^−1^, P-O at 1091 cm^−1^, Ca-O at 1047 cm^−1^, and O-Ca-O at 1031 cm^−1^ [64,65,80].

It should be noted that the splitting of the main phosphate band ν_3_ PO_4_ in IR spectra is often associated with the substitution of calcium atoms in the crystal lattice by external ions. In this case, a characteristic feature is the appearance of a band in the IR spectra in the region of 1095 cm^−1^, assigned to the Ca-O-P bond [81], which is also observed in our experimental spectrum.

Octocalcium phosphate present in solid tissues is often used to represent the structure of defective calcium hydroxyapatite as well as biogenic apatites. Rey et al. in [74] showed in detail that the spectrum of octocalcium phosphate contains several overlapping high intensity bands around 1103 cm^−1^, 1077 cm^−1^, 1055 cm^−1^, 1037 cm^−1^, and 1023 cm^−1^, whereas stoichiometric hydroxyapatite is characterized by oscillations around 1092 cm^−1^ and 1040 cm^−1^. This fact indicates that a change in the local environment of calcium atoms leads to the transformation of vibrational spectra and the appearance of additional bands. This reasoning is confirmed by the calculated data from [78].

It should be noted that the vibrational bands described in [77,78] have already been recorded by Raman spectroscopy in our previous work devoted to studies of enamel, dentin, and calcium hydroxyapatite [45]. In this case, the calculated values of frequencies for Ca-O bonds in the far-wavelength region of the Raman spectrum are in excellent agreement with the experiment [41,82,83].

Thus, the results of the theoretical analysis [77,78] confirm the experimental data on the vibrational spectrum of calcium phosphates [56,74,75,76], indicating the features in the IR spectra (reflections and absorptions) associated with changes in the local environment of calcium atoms in phosphates with different stoichiometry. Therefore, taking into account the classification of possible bonds described above, the experimental profile of the main phosphate band ν_3_ PO_4_ in the IR reflectance spectra (Figure 4) seems to be the result of the overlap of P-O, O-P-O, O-Ca-O, and Ca-O-P vibrational modes.

In this connection, based on the approach tested by us in a number of papers [84,85], we performed deconvolution of IR spectral profiles for C1-C4 clusters. The decomposition was performed taking into account the limitations imposed on the procedure for determining the number of maxima in the spectrum, calculating and removing the baseline, and determining the convergence of the decomposition result. 

**Figure 5:**
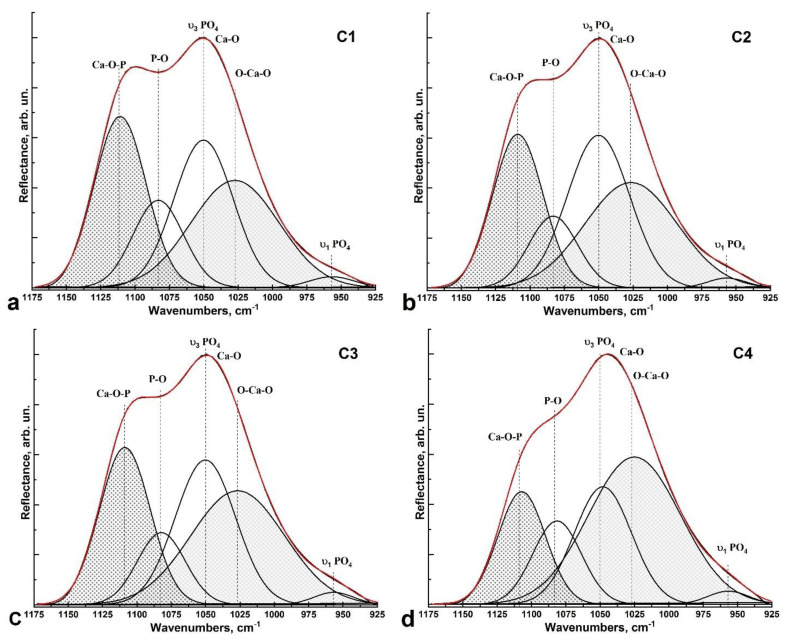
Deconvolution of the ν_3_ PO_4_^3−^ vibrational band profile in the spectra: (**a**) cluster C1 (intact enamel region); (**b**,**c**) clusters C2 and C3 from the zone near the enamel-side hybrid interface; (**d**) cluster C4 (PDA hybrid interface zone).

The results of the deconvolution of the spectral profiles for the C1 to C4 clusters are presented in Figure 5. Table 1 compiles the frequencies (centers of gravity), full width at half maximum (FWHM), and intensities of phonon modes, as well as their belonging to vibrations of characteristic molecular groups.

Deconvolution analysis shows that as the interface (PDA hybrid layer C4) approaches the healthy enamel side (C1), the intensities, FWHM, and frequency positions of the O-Ca-O and Ca-O-P vibrations change significantly (Figure 5a–d). The frequencies of both maxima decrease, with a low-frequency shift of ~5 cm^−1^ for the Ca-O-P mode. While the intensity of the oscillation associated with the Ca-O-P bonds decreases, the intensity of the O-Ca-O mode increases proportionally. The change in vibration intensity as well as the frequency shift is related both to phase transformations occurring in the area of pre-treated dental tissue under the influence of the diamond micro drill, and the subsequent pretreatment performed (etching and leaching). The increase in O-Ca-O bonds also indicates a significant increase in Ca^2+^ calcium ions in the boundary area and in the hybrid layer, which is a consequence of cavity treatment with Ca(OH)_2_ solution.

It should be noted that Raman and FTIR microspectroscopy confirm the formation of a PDA hybrid layer between the natural enamel and dental composite. However, at the same time, high-resolution FTIR microspectroscopy, due to the greater depth of analysis, gives the average characteristics of the entire structure. Raman spectroscopy, despite the fact that the analyzed spectra are averaged from similar areas for a series of similar samples, is still a local method of analysis, and reveals subtle features. Both methods capture changes in the molecular composition occurring at the interface during the formation of the PDA hybrid layer.

Thus, a comprehensive analysis of the PDA coating technology and identified molecular composition features in the enamel/polydopamine/composite interface (Figure 1 and Figure 3) suggests that polydopamine layer deposition occurs over the entire surface of the cavity formed in the enamel. The accelerated deposition conditions of the PDA film used in this work (see methodology) make it possible to obtain layers covering the surface evenly (Figure 1 and Figure 3), taking into account its macro- and microrelief features, which should contribute to the formation of a better adhesion with restorative dental materials, as was predicted earlier [21]. It has been previously noted that PDA can be used to implement the mechanism of mineralization of dental tissue and improve the adsorption and adhesion of various agents on the surface of the materials used for restoration of dental tissue [86,87]. For example, a functional material synthesized on the basis of HEMA adhesive with PDA, nanosized hydroxyapatite, and silver particles with antibacterial activity showed improved adhesion characteristics in dental restorations. Jiahui Zhang et al. [87] pointed out that the catecholamine groups of PDA introduced into a functional adhesive can combine with Ca^2+^. In this case, PDA enriches and promotes the formation of calcium phosphates [87] and induces the formation of hydroxyapatite on its surface [88]. At the same time, it has been shown that the introduction of nanocrystalline hydroxyapatite as a filler in the matrix of a universal web-curing adhesive can achieve increased hardness values and an increased degree of conversion during the photopolymerization of modified adhesives [89,90,91]. This is due to the interaction of nanocrystalline hydroxyapatite with the molecular groups of the adhesive, as well as a change in the chemical bonds in the system [89]. At the same time, as was rightly noted in [87], the bonding strength should not be reduced by the introduction of functional fillers to modify the adhesive. In contrast to Jiahui Zhang et al. [87], in our current work, only polydopamine deposited on the pre-treated enamel surface was used as an adhesive in the formation of the hybrid layer. The use of calcium hydroxide Ca(OH)_2_ allowed stable calcium orthophosphates to be introduced into the hybrid layer, with an increased content of Ca ions. The catecholamine groups of polydopamine can simultaneously bind to both Ca^2+^ apatite and O-Ca-O groups from the hybrid layer, thereby improving the quality of the restoration.

In this way, a homogeneous bioinspired PDA–hybrid interface between dental composites and natural dental tissues can be formed using a sufficiently simple dopamine polymerization process and an alkalinization procedure.

## 3. Materials and Methods

### 3.1. Design of Experiment

Healthy teeth (without carious lesions or erosions) extracted for orthodontic indications from donors aged 20–25 years were selected for our studies. Teeth were collected in accordance with relevant guidelines and regulations. After removal, the samples were placed in individual vials containing 0.9% saline and 0.002% sodium azide, and stored at 4 °C.

Ten teeth were used to form the hybrid layer and reproduce the synthesis conditions. Dental tissue samples were prepared according to standard procedures, the requirements of manufacturers of dental bonding systems, and the conditions necessary to obtain polydopamine-based nanolayers.

Initially, a cylindrical cavity of 3 × 3 mm was formed in each tooth orthogonal to the occlusal surface. A diamond cylindrical micro drill with a turbine handpiece and constant water jet cooling were used for this purpose. In order to avoid overheating of the dental hard tissue, the cavity walls were formed at low rotational speeds of the diamond micro drill.

The formed cavity was then rinsed with distilled water and treated with a 38% phosphoric acid H_3_PO_4_ solution to remove the lubricated layer, form a micro-relief, and functionalize the surface according to standard enamel preparation procedures [5]. After 30 s, the cavity surface was rinsed twice with distilled water to remove residual etching products and dried.

As previously shown, the use of an acidic environment during treatment can negatively affect material adhesion and affect the hydroxyapatite–organic complex bond [4,92,93,94,95]. Therefore, after using phosphoric acid, the cavity was treated with a 0.016% solution of calcium hydroxide Ca(OH)_2_ for 30 s. This procedure was performed to reduce the formation of metastable calcium orthophosphates on the surface of pretreated enamel, which have increased solubility and reduce the stability of the formed hybrid layer. The increased calcium content of this treatment promotes both the formation of more stable forms of calcium phosphates and the maintenance of an alkaline pH value in the interface zone, which is required to obtain polydopamine-based coatings [34].

The next step was the deposition of the polydopamine film. The procedure was performed in two stages according to the accelerated deposition mechanism. At the first stage, dental tissue samples were placed in a solution containing dopamine hydrochloride with a concentration of 2 mg/mL. After that, 5 mM CuSO_4_•6H_2_O solution was added to this solution at a constant stirring speed of 200 rpm. Next, 20 mM H_2_O_2_ was added to this solution to produce so-called reactive ionic complexes, which contribute to the accelerated oxidation of polydopamine [34]. After 1 h, the dental tissue samples were washed twice with a stream of distilled water.

It has been shown previously that after a single deposition of polydopamine using the described technology, a uniform film is formed, regardless of the surface microrelief [34]. At the same time, polydopamine deposition depends on the state of charge complexes on the deposited surface and, if uncompensated charges are present on it, the polydopamine film may be deposited non-uniformly [32]. It is also known that enamel apatite nanocrystals have uncompensated charges on their surface. Moreover, the hierarchical structure of the enamel cores and the interstitial space will set the nonhomogeneous deposition of the polydopamine film on the surface of the formed cavity. Taking these factors into account, the second stage of the accelerated deposition of the polydopamine film was carried out. For this purpose, the samples were also placed in a solution containing dopamine hydrochloride with a concentration of 2 mg/mL, and 5 mM CuSO_4_•6H_2_O and 60 mM H_2_O_2_ were added at a constant stirring speed of 300 rpm. Due to the higher concentration of the oxidant, the rate of deposition of the PDA film also increased, so the time for the second step was 30 min.

After the second stage of polydopamine film deposition, the samples were washed twice in distilled water and then dried with compressor air flow. In the last step, Synergy D6 flow commercial material (Manufacturer: Coltène/Whaledent; Type: Microhybrid flowable; Resin matrix: Methacrylates; Filler: Silanized barium glass, hydrophobic amorphous silica) [96] was applied according to the manufacturer’s instructions and photopolymerized for 60 s.

### 3.2. Sectioning of Dental Tissue

To conduct studies of the hybrid interface formed according to the described technology using microspectroscopic methods of analysis, the tooth samples were divided into 1 mm thick segments using a water-cooled diamond low-speed saw. Each segment was polished using diamond paste. After this procedure, the tooth samples were placed in sealed containers with constant humidity, where they were stored until structural spectroscopic studies were performed [25,45].

### 3.3. Synthesis of Dopamine Hydrochloride

**Scheme 1:**
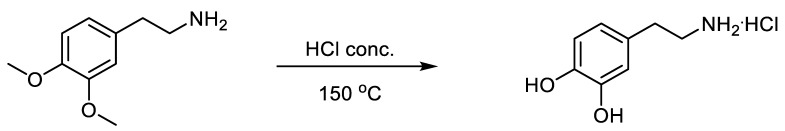
Syntes of dopamine hydrochloride.

For our studies, dopamine hydrochloride was obtained under laboratory conditions according to the following Scheme 1.

A mixture of 1.81 g of 2-(3,4-dimethoxyphenyl)ethane-1-amine and 10 mL of concentrated hydrochloric acid was heated in a sealed ampoule at 150 °C for two hours. The resulting mass was evaporated to a dry residue, and the residue was purified by preparative column chromatography on silica gel (60 µm) with methanol as eluent. After evacuation of the methanol, the residue was recrystallized from 20% hydrochloric acid. The yield was 1.35 g (71%), m. p. 238–239 °C. ^1^H NMR spectrum δ, ppm; J, Hz: 2.67–2.72 (2H, m, CH_2_); 2.90–2.93 (2H, m, CH_2_); 3.41–3.47 (3H + H_2_O, m, NH_2_·HCl); 6.47 (1H, dd, J_1_ = 8.0, J_2_ = 2.1, C(6)H-arom); 6.63 (1H, d, J = 2.1, C(2)H-arom); 6.68 (1H, d, J = 8.0, C(5)H-arom); 8.03 (2H, br.s, 2OH). Mass spectrum: found *m/z*, 154.086 [M + H]^+^, calculated for C_8_H_12_NO_2_^+^, 154.0863.

The ^1^H NMR spectrum was recorded on a Bruker DRX500 (500 MHz) in DMSO-d_6_, internal standard—TMS. The high-resolution mass spectrum was recorded on an Agilent Technologies LCMS TOF 6230B instrument, with a dual-ESI ionization method in a nitrogen atmosphere. Separation conditions: mobile phase 0.1% formic acid in MeCN (eluent A)/0.1% formic acid in water (eluent B). Gradient: start A:B = 60:40% for 1 min, in 3.5 min 60–100% A, then 100% A. Flow 0.4 mL/min, column—Poroshell 120 EC-C18 (4.6 × 50 mm, 2.7 μm), thermostat 28 °C, electrospray ionization (capillary −3.5 kV; fragment +191 V; OctRF +66 V—positive polarity). Melting temperatures were determined using a Stuart SMP30 instrument.

### 3.4. Experimental Set-Up and Parameters

#### 3.4.1. Optical Microscopy

Optical images of the interface areas were collected using an Olympus CX40 optical microscope. Olympus 40×/0.65 Planachromatic objective and Levenhuk 5 MPix digital camera were used. The images were obtained in light field mode with flat field correction. The size of the area on the surface of the samples falling into the lens was 300 × 400 μ^2^ at a magnification of ×600. The images were analyzed using the TaupTek TaupVeiw version 3.7 software package (Hangzhou ToupTek Photonics Co., Hangzhou, China).

#### 3.4.2. Raman Microspectroscopy

Point-spectral analysis of the molecular composition in the area of PDA hybrid interface between the tooth tissue and dental composite was conducted via Raman microspectroscopy. Spectra were obtained using a confocal Raman microscope RamMix 532 (InSpectr, Moscow, Russia) with 532 nm wavelength laser excitation and 25 mW of radiation power at the sample. The spectrum was collected in the range of 800–2000 cm^−1^ with a spectral resolution of 1 cm^−1^. The choice of the microregions in the area of the PDA–hybrid interface was made using an automated motorized 2-axis stage. Signals from the surface of the sample were collected with a 100× objective.

MicroRaman spectral data pre-processing, baseline correction, averaging, maximum positioning, integral area values, and decomposition into components were performed using Origin 8 software (OriginLab Corporation, Northampton, MA, USA). 

#### 3.4.3. High-Resolution FTIR Hyperspectral Analysis

FTIR spectral imaging of the interface sections was carried out at the IMBUIA beamline of the fourth-generation storage ring Sirius, at the Brazilian Synchrotron Light Laboratory (Campinas, Brazil), using an Agilent Cary 660 FTIR microscope. High-sensitivity spectral analysis and full-field spectral-imaging were performed using a 128 × 128 Focal Plane Array (FPA) IR detector (MCT type) in reflection mode. Spectra were collected in the 1800–900 cm^−1^ region with a spectral resolution of at least 3.5 cm^−1^, using a 25× high objective with high magnification mode. A region of 60 × 60 μm^2^ with an effective pixel size of 0.66 μm^2^ was analyzed.

Background spectra were acquired on the clean surface of a 100 nm thick gold film sputtered on silicon, which was placed next to the teeth sample, using 256 co-added scans. Blackman–Harris three-term apodization, Mertz phase correction, and a zero-filling factor of 2 were set as the default acquisition parameters, using Agilent Resolutions Pro version 5.0 software.

The processing of the collected hyperspectral dataset (normalization, smoothing, baseline removal, chemical mapping, and multivariate spectral analysis) was performed using CytoSpec v. 2.00.07.

#### 3.4.4. Hyperspectral Data Processing

In our work, the second derivative and vector normalization in the 2000–900 cm^−1^ region of the IR spectrum were used to process the initial spectral data in our hierarchical cluster analysis (HCA), as the main vibrations characteristic of the interface zone chemicals are located there. Smoothing of the original IR spectra was performed at nine points.

The best cluster analysis algorithm was selected experimentally. The Euclidean distance was chosen as the proximity function. For clustering, Ward’s method was applied, which generates multiple partitions of the original image and considers all combinations of clusters, i.e., groups of spectra according to similarity, using variance increase as a function of distance [97]. The HCA and surface chemical images were created using CytoSpec v. 2.00.07 software.

The procedure for deconvolution of the spectra into its components, including determination of the range of local extrema (the minima and maxima of spectral function) and overlapping frequencies, was performed using Origin 8 software. The range of local extrema was determined on the basis of the calculation of the second and fourth derivatives. The quality of the expansion was determined with reference to the error minimization criterion (χ-square). As a result, the necessary criterion of convergence and reproducibility of the simulation results was found, which ensured unambiguous decomposition.

## 4. Conclusions

By combining microscopic and spectral analysis techniques with multidimensional hyperspectral array processing techniques, this paper shows that a PDA hybrid interface between natural hard dental enamel and dental composite can be formed using the proposed synthesis technology.

It was found that traditional methods of pre-treatment of dental tissue create a micrometric relief of the enamel/dental composite interface surface, within which (~4 μ) the spatial distribution of the polydopamine film, acting like a natural adhesive and providing adhesion of the materials, is detected. Even though natural enamel has spatial variations in chemical and phase composition (and its pretreatment at the stage of dental restorations not only reduces the degree of crystallinity, but also changes the morphology of the boundary layer and the phase composition in the micron region), the performed procedure of leaching and the subsequent deposition of bioinspired PDA adhesive allowed the formation of a hybrid interface with increased O-Ca-O bond content.

The possibility of forming PDA on the dental surface can help to restore lost mineral complexes in preconditioned hard tissue, and can improve the quality of the restoration by creating a hybrid interface without composite resin bonding, which is known to be the weak link in restorations [86,87].

The creation of PDA-based bionanocomposites in the form of buffer (hybrid) layers providing improved adhesion to two heterogeneous materials is a promising direction in the field of dental materials science. However, the introduction of the developed technologies into clinical practice for minimally invasive dental restoration procedures requires more detailed study and approbation of the technology of accelerated deposition of PDA hybrid layers, the creation of self-assembled biomimetic nanocomposites with antibacterial properties, and more study of aging processes (including conditions close to those of real life).

## Figures and Tables

**Table 1:** Parameters of the Gaussian curve components used in the decomposition of the ν_3_ PO_4_^3−^ spectral band in the 1175–925 cm^−1^ region.

Band	Center of Gravity, cm^−1^				FWHM, cm^−1^				Intens. of Peak, arb.un.			
	C1	C2	C3	C4	C1	C2	C3	C4	C1	C2	C3	C4
ν_1_ PO_4_^3−^	957	957	957	957	39	33	34	35	4	4	5	5
O-Ca-O	1027	1027	1027	1025	76	80	81	79	43	42	46	59
ν_3_ PO_4_^3−^	1050	1050	1050	1048	51	53	52	49	59	61	58	47
P-O	1083	1083	1082	1081	43	42	41	42	35	29	29	33
Ca-O-P	1111	1109	1109	1107	45	44	44	41	69	62	63	45

## Data Availability

The data that support the findings of this study are available from the corresponding author upon reasonable request.

## References

[1] Pandya M., Diekwisch T.G.H. (2019). Enamel Biomimetics—Fiction or Future of Dentistry. Int. J. Oral Sci..

[2] Riau A.K., Venkatraman S.S., Mehta J.S. (2020). Biomimetic vs. Direct Approach to Deposit Hydroxyapatite on the Surface of Low Melting Point Polymers for Tissue Engineering. Nanomaterials.

[3] Wang J., Liu Z., Ren B., Wang Q., Wu J., Yang N., Sui X., Li L., Li M., Zhang X. (2021). Biomimetic Mineralisation Systems for in Situ Enamel Restoration Inspired by Amelogenesis. J. Mater. Sci. Mater. Med..

[4] Seredin P., Goloshchapov D., Kashkarov V., Emelyanova A., Buylov N., Barkov K., Ippolitov Y., Khmelevskaia T., Mahdy I.A., Mahdy M.A. (2022). Biomimetic Mineralization of Tooth Enamel Using Nanocrystalline Hydroxyapatite under Various Dental Surface Pretreatment Conditions. Biomimetics.

[5] Sato T., Takagaki T., Hatayama T., Nikaido T., Tagami J. (2021). Update on Enamel Bonding Strategies. Front. Dent. Med..

[6] Tampieri A., Iafisco M., Sprio S., Ruffini A., Panseri S., Montesi M., Adamiano A., Sandri M., Antoniac I.V. (2016). Hydroxyapatite: From Nanocrystals to Hybrid Nanocomposites for Regenerative Medicine. Handbook of Bioceramics and Biocomposites.

[7] Fang Z., Guo M., Zhou Q., Li Q., Wong H.M., Cao C.Y. (2021). Enamel-like Tissue Regeneration by Using Biomimetic Enamel Matrix Proteins. Int. J. Biol. Macromol..

[8] Mohabatpour F., Chen X., Papagerakis S., Papagerakis P. (2022). Novel Trends, Challenges and New Perspectives for Enamel Repair and Regeneration to Treat Dental Defects. Biomater. Sci..

[9] Chang R., Liu Y.-J., Zhang Y.-L., Zhang S.-Y., Han B.-B., Chen F., Chen Y.-X. (2022). Phosphorylated and Phosphonated Low-Complexity Protein Segments for Biomimetic Mineralization and Repair of Tooth Enamel. Adv. Sci..

[10] Ding L., Han S., Wang K., Zheng S., Zheng W., Peng X., Niu Y., Li W., Zhang L. (2020). Remineralization of Enamel Caries by an Amelogenin-Derived Peptide and Fluoride in Vitro. Regen. Biomater..

[11] Heintze S.D., Loguercio A.D., Hanzen T.A., Reis A., Rousson V. (2022). Clinical Efficacy of Resin-Based Direct Posterior Restorations and Glass-Ionomer Restorations—An Updated Meta-Analysis of Clinical Outcome Parameters. Dent. Mater..

[12] Perdigão J. (2020). Current Perspectives on Dental Adhesion: (1) Dentin Adhesion—Not There Yet. Jpn. Dent. Sci. Rev..

[13] Betancourt D.E., Baldion P.A., Castellanos J.E. (2019). Resin-Dentin Bonding Interface: Mechanisms of Degradation and Strategies for Stabilization of the Hybrid Layer. Int. J. Biomater..

[14] Mota A.L.M., Macedo F.A.A., Lemos M.V.S., Mendes T.A.D., Lourenço G.A., Albuquerque N.L.G., Feitosa V.P., Santiago S.L. (2018). Evaluation of Dentinal Conditioning with Natural Acids in Dentin-Resin Interface. Dent. Mater..

[15] Nicholson J., Czarnecka B. (2016). Materials for the Direct Restoration of Teeth.

[16] Vinagre A., Ramos J. (2016). Adhesion in Restorative Dentistry.

[17] Lempel E., Lovász B.V., Bihari E., Krajczár K., Jeges S., Tóth Á., Szalma J. (2019). Long-Term Clinical Evaluation of Direct Resin Composite Restorations in Vital vs. Endodontically Treated Posterior Teeth—Retrospective Study up to 13 Years. Dent. Mater..

[18] Ball V. (2017). Composite Materials and Films Based on Melanins, Polydopamine, and Other Catecholamine-Based Materials. Biomimetics.

[19] Coy E., Iatsunskyi I., Colmenares J.C., Kim Y., Mrówczyński R. (2021). Polydopamine Films with 2D-like Layered Structure and High Mechanical Resilience. ACS Appl. Mater. Interfaces.

[20] Liu Y., Ai K., Lu L. (2014). Polydopamine and Its Derivative Materials: Synthesis and Promising Applications in Energy, Environmental, and Biomedical Fields. Chem. Rev..

[21] Jia L., Han F., Wang H., Zhu C., Guo Q., Li J., Zhao Z., Zhang Q., Zhu X., Li B. (2019). Polydopamine-Assisted Surface Modification for Orthopaedic Implants. J. Orthop. Transl..

[22] Alfieri M.L., Panzella L., Oscurato S.L., Salvatore M., Avolio R., Errico M.E., Maddalena P., Napolitano A., D’Ischia M. (2018). The Chemistry of Polydopamine Film Formation: The Amine-Quinone Interplay. Biomimetics.

[23] Devarajan S.S., Madhubala M.M., Rajkumar K., James V., Srinivasan N., Mahalaxmi S., Sathyakumar S. (2021). Effect of Polydopamine Incorporated Dentin Adhesives on Bond Durability. J. Adhes. Sci. Technol..

[24] James V., Madhubala M.M., Devarajan S.S., Mahalaxmi S., Sathyakumar S. (2020). Evaluation of Degree of Conversion, Resin-Dentin Bond Strength, and Durability of Polydopamine Incorporated Total Etch Adhesive System. Front. Dent..

[25] Seredin P., Goloshchapov D., Ippolitov Y., Vongsvivut J. (2021). Engineering of a Biomimetic Interface between a Native Dental Tissue and Restorative Composite and Its Study Using Synchrotron FTIR Microscopic Mapping. Int. J. Mol. Sci..

[26] Vongsvivut J., Pérez-Guaita D., Wood B.R., Heraud P., Khambatta K., Hartnell D., Hackett M.J., Tobin M.J. (2019). Synchrotron Macro ATR-FTIR Microspectroscopy for High-Resolution Chemical Mapping of Single Cells. Analyst.

[27] Querido W., Kandel S., Pleshko N. (2021). Applications of Vibrational Spectroscopy for Analysis of Connective Tissues. Molecules.

[28] Ye Q., Spencer P. (2017). Analyses of Material-Tissue Interfaces by Fourier Transform Infrared, Raman Spectroscopy, and Chemometrics. Material-Tissue Interfacial Phenomena.

[29] Shah F.A. (2019). Micro-Raman Spectroscopy Reveals the Presence of Octacalcium Phosphate and Whitlockite in Association with Bacteria-Free Zones Within the Mineralized Dental Biofilm. Microsc. Microanal..

[30] Wang P., Anderson E.J.D., Muller E.A., Gao F., Zhong Y., Raschke M.B. (2018). Hyper-Spectral Raman Imaging Correlating Chemical Substitution and Crystallinity in Biogenic Hydroxyapatite: Dentin and Enamel in Normal and Hypoplastic Human Teeth. J. Raman Spectrosc..

[31] Seredin P., Goloshchapov D., Kashkarov V., Khydyakov Y., Nesterov D., Ippolitov I., Ippolitov Y., Vongsvivut J. (2022). Development of a Hybrid Biomimetic Enamel-Biocomposite Interface and a Study of Its Molecular Features Using Synchrotron Submicron ATR-FTIR Microspectroscopy and Multivariate Analysis Techniques. Int. J. Mol. Sci..

[32] Bogdan D., Grosu I.-G., Filip C. (2022). How Thick, Uniform and Smooth Are the Polydopamine Coating Layers Obtained under Different Oxidation Conditions? An in-Depth AFM Study. Appl. Surf. Sci..

[33] Salomäki M., Marttila L., Kivelä H., Ouvinen T., Lukkari J. (2018). Effects of PH and Oxidants on the First Steps of Polydopamine Formation: A Thermodynamic Approach. J. Phys. Chem. B.

[34] Zhang C., Ou Y., Lei W.-X., Wan L.-S., Ji J., Xu Z.-K. (2016). CuSO_4_/H_2_O_2_-Induced Rapid Deposition of Polydopamine Coatings with High Uniformity and Enhanced Stability. Angew. Chem. Int. Ed..

[35] Jegova G., Titorenkova R., Rashkova M., Mihailova B. (2013). Raman and IR Reflection Micro-Spectroscopic Study of Er:YAG Laser Treated Permanent and Deciduous Human Teeth. J. Raman Spectrosc..

[36] Shamsudeen S.M., Thavarajah R., Joshua E., Rao U.D.K., Kannan R. (2019). Evaluating and Comparing the Morphological and Histopathological Changes Induced by Erbium:Yttrium-Aluminum-Garnet Laser and Diamond Bur on Enamel, Dentin and Pulp Tissue. J. Investig. Clin. Dent..

[37] Penel G., Leroy G., Rey C., Bres E. (1998). MicroRaman Spectral Study of the PO_4_ and CO_3_ Vibrational Modes in Synthetic and Biological Apatites. Calcif. Tissue Int..

[38] Mihály J., Gombás V., Afishah A., Mink J. (2009). FT-Raman Investigation of Human Dental Enamel Surfaces. J. Raman Spectrosc..

[39] Spizzirri P.G., Cochrane N.J., Prawer S., Reynolds E.C. (2012). A Comparative Study of Carbonate Determination in Human Teeth Using Raman Spectroscopy. Caries Res..

[40] Zelic K., Milovanovic P., Rakocevic Z., Askrabic S., Potocnik J., Popovic M., Djuric M. (2014). Nano-Structural and Compositional Basis of Devitalized Tooth Fragility. Dent. Mater. Off. Publ. Acad. Dent. Mater..

[41] Bērziņš K., Sutton J.J., Loch C., Beckett D., Wheeler B.J., Drummond B.K., Fraser-Miller S.J., Gordon K.C. (2019). Application of Low-wavenumber Raman Spectroscopy to the Analysis of Human Teeth. J. Raman Spectrosc..

[42] Pezzotti G., Zhu W., Boffelli M., Adachi T., Ichioka H., Yamamoto T., Marunaka Y., Kanamura N. (2015). Vibrational Algorithms for Quantitative Crystallographic Analyses of Hydroxyapatite-Based Biomaterials: I, Theoretical Foundations. Anal. Bioanal. Chem..

[43] Sowa M.G., Popescu D.P., Werner J., Hewko M., Ko A.C.-T., Payette J., Dong C.C.S., Cleghorn B., Choo-Smith L.-P. (2006). Precision of Raman Depolarization and Optical Attenuation Measurements of Sound Tooth Enamel. Anal. Bioanal. Chem..

[44] Goloshchapov D.L., Lenshin A.S., Savchenko D.V., Seredin P.V. (2019). Importance of Defect Nanocrystalline Calcium Hydroxyapatite Characteristics for Developing the Dental Biomimetic Composites. Results Phys..

[45] Goloshchapov D., Buylov N., Emelyanova A., Ippolitov I., Ippolitov Y., Kashkarov V., Khudyakov Y., Nikitkov K., Seredin P. (2021). Raman and XANES Spectroscopic Study of the Influence of Coordination Atomic and Molecular Environments in Biomimetic Composite Materials Integrated with Dental Tissue. Nanomaterials.

[46] Sadyrin E., Swain M., Mitrin B., Rzhepakovsky I., Nikolaev A., Irkha V., Yogina D., Lyanguzov N., Maksyukov S., Aizikovich S. (2020). Characterization of Enamel and Dentine about a White Spot Lesion: Mechanical Properties, Mineral Density, Microstructure and Molecular Composition. Nanomaterials.

[47] Choo-Smith L.-P., Hewko M., Sowa M.G., Matousek P., Morris M.D. (2010). Emerging Dental Applications of Raman Spectroscopy. Emerging Raman Applications and Techniques in Biomedical and Pharmaceutical Fields.

[48] Ko A.C.-T., Choo-Smith L.-P., Hewko M., Sowa M.G., Dong C.C.S., Cleghorn B. (2006). Detection of Early Dental Caries Using Polarized Raman Spectroscopy. Opt. Express.

[49] Wang Z., Zheng W., Hsu S.C.-Y., Huang Z. (2016). Optical Diagnosis and Characterization of Dental Caries with Polarization-Resolved Hyperspectral Stimulated Raman Scattering Microscopy. Biomed. Opt. Express.

[50] Timchenko E.V., Bazhutova I.V., Frolov O.O., Volova L.T., Timchenko P.E. (2021). Raman Spectroscopy for Assessment of Hard Dental Tissues in Periodontitis Treatment. Diagnostics.

[51] Desoutter A., Slimani A., Al-Obaidi R., Barthélemi S., Cuisinier F., Tassery H., Salehi H. (2019). Cross Striation in Human Permanent and Deciduous Enamel Measured with Confocal Raman Microscopy. J. Raman Spectrosc..

[52] Al-Obaidi R., Salehi H., Collart-Dutilleul P.-Y., Jacquot B., Tassery H., Cuisinier F.J.G., Gergely C., Cloitre T. (2020). Relationship between Changes in Chemical Composition of Enamel Subsurface Lesions and the Emitted Nonlinear Optical Signals: An in Vitro Study. Caries Res..

[53] Seredin P., Goloshchapov D., Ippolitov Y., Vongsvivut J. (2020). Development of a New Approach to Diagnosis of the Early Fluorosis Forms by Means of FTIR and Raman Microspectroscopy. Sci. Rep..

[54] Campillo M., Lacharmoise P.D., Reparaz J.S., Goñi A.R., Valiente M. (2010). On the Assessment of Hydroxyapatite Fluoridation by Means of Raman Scattering. J. Chem. Phys..

[55] Awonusi A., Morris M.D., Tecklenburg M.M.J. (2007). Carbonate Assignment and Calibration in the Raman Spectrum of Apatite. Calcif. Tissue Int..

[56] Taylor E.A., Donnelly E. (2020). Raman and Fourier Transform Infrared Imaging for Characterization of Bone Material Properties. Bone.

[57] Montes-Hernandez G., Renard F. (2020). Nucleation of Brushite and Hydroxyapatite from Amorphous Calcium Phosphate Phases Revealed by Dynamic In Situ Raman Spectroscopy. J. Phys. Chem. C.

[58] Kim D.-H., Hwang K.-H., Lee J.D., Park H.-C., Yoon S.-Y. (2015). Long and Short Range Order Structural Analysis of In-Situ Formed Biphasic Calcium Phosphates. Biomater. Res..

[59] Seredin P., Goloshchapov D., Buylov N., Kashkarov V., Emelyanova A., Eremeev K., Ippolitov Y. (2022). Compositional Analysis of the Dental Biomimetic Hybrid Nanomaterials Based on Bioinspired Nonstoichiometric Hydroxyapatite with Small Deviations in the Carbonate Incorporation. Nanomaterials.

[60] Mallinson D., Mullen A.B., Lamprou D.A. (2018). Probing Polydopamine Adhesion to Protein and Polymer Films: Microscopic and Spectroscopic Evaluation. J. Mater. Sci..

[61] Perna G., Lasalvia M., Capozzi V. (2016). Vibrational Spectroscopy of Synthetic and Natural Eumelanin. Polym. Int..

[62] Khan A.S., Khalid H., Sarfraz Z., Khan M., Iqbal J., Muhammad N., Fareed M.A., Rehman I.U. (2017). Vibrational Spectroscopy of Selective Dental Restorative Materials. Appl. Spectrosc. Rev..

[63] Wang Y., Yao X., Parthasarathy R. (2009). Characterization of Interfacial Chemistry of Adhesive/Dentin Bond Using FTIR Chemical Imaging with Univariate and Multivariate Data Processing. J. Biomed. Mater. Res. A.

[64] Diez-García S., Sánchez-Martín M.-J., Amigo J.M., Valiente M. (2022). Combination of Two Synchrotron Radiation-Based Techniques and Chemometrics to Study an Enhanced Natural Remineralization of Enamel. Anal. Chem..

[65] Babot-Marquillas C., Sánchez-Martín M.-J., Amigo J.M., Yousef I., Valido I.H., Boada R., Valiente M. (2022). Tooth Whitening, Oxidation or Reduction? Study of Physicochemical Alterations in Bovine Enamel Using Synchrotron Based Micro-FTIR. Dent. Mater..

[66] Zangmeister R.A., Morris T.A., Tarlov M.J. (2013). Characterization of Polydopamine Thin Films Deposited at Short Times by Autoxidation of Dopamine. Langmuir.

[67] Beniash E., Stifler C.A., Sun C.-Y., Jung G.S., Qin Z., Buehler M.J., Gilbert P.U.P.A. (2019). The Hidden Structure of Human Enamel. Nat. Commun..

[68] Teaford M.F., Smith M.M., Ferguson M.W.J. (2007). Development, Function and Evolution of Teeth.

[69] Han F., Liang R., Xie H. (2021). Effects of Phosphoric Acid Pre-Etching on Chemisorption between Enamel and MDP-Containing Universal Adhesives: Chemical and Morphological Characterization, and Evaluation of Its Potential. ACS Omega.

[70] Seredin P.V., Goloshchapov D.L., Prutskij T., Ippolitov Y.A. (2017). Fabrication and Characterisation of Composites Materials Similar Optically and in Composition to Native Dental Tissues. Results Phys..

[71] Lopes C.d.C.A., Limirio P.H.J.O., Novais V.R., Dechichi P. (2018). Fourier Transform Infrared Spectroscopy (FTIR) Application Chemical Characterization of Enamel, Dentin and Bone. Appl. Spectrosc. Rev..

[72] Reyes-Gasga J., Martínez-Piñeiro E.L., Rodríguez-Álvarez G., Tiznado-Orozco G.E., García-García R., Brès E.F. (2013). XRD and FTIR Crystallinity Indices in Sound Human Tooth Enamel and Synthetic Hydroxyapatite. Mater. Sci. Eng. C.

[73] Jeon R.J., Hellen A., Matvienko A., Mandelis A., Abrams S.H., Amaechi B.T. (2008). In Vitro Detection and Quantification of Enamel and Root Caries Using Infrared Photothermal Radiometry and Modulated Luminescence. J. Biomed. Opt..

[74] Rey C., Marsan O., Combes C., Drouet C., Grossin D., Sarda S. (2014). Characterization of Calcium Phosphates Using Vibrational Spectroscopies. Advances in Calcium Phosphate Biomaterials.

[75] Karampas I.A., Kontoyannis C.G. (2013). Characterization of Calcium Phosphates Mixtures. Vib. Spectrosc..

[76] Berzina-Cimdina L., Borodajenko N., Theophile T. (2012). Research of Calcium Phosphates Using Fourier Transform Infrared Spectroscopy. Infrared Spectroscopy—Materials Science, Engineering and Technology.

[77] Khavryuchenko V.D., Khavryuchenko O.V., Lisnyak V.V. (2007). Quantum Chemical and Spectroscopic Analysis of Calcium Hydroxyapatite and Related Materials. J. Solid State Chem..

[78] Lin T.-J., Chiu C.-C. (2017). Structures and Infrared Spectra of Calcium Phosphate Clusters by Ab Initio Methods with Implicit Solvation Models. Phys. Chem. Chem. Phys..

[79] Bhat P.A., Debnath N.C. (2013). Study of Structures and Properties of Silica-Based Clusters and Its Application to Modeling of Nanostructures of Cement Paste by DFT Methods. IOP Conf. Ser. Mater. Sci. Eng..

[80] Diez García S. (2021). In Vitro Natural Remineralization of Enamel: Characterization by Conventional and Synchrotron Radiation-Based Techniques. Ph.D. Thesis.

[81] Basu S., Nag S., Kottan N.B., Basu B. (2022). In Silico Study on Probing Atomistic Insights into Structural Stability and Tensile Properties of Fe-Doped Hydroxyapatite Single Crystals. Sci. Rep..

[82] Antonakos A., Liarokapis E., Leventouri T. (2007). Micro-Raman and FTIR Studies of Synthetic and Natural Apatites. Biomaterials.

[83] Calderín L., Dunfield D., Stott M.J. (2005). Shell-Model Study of the Lattice Dynamics of Hydroxyapatite. Phys. Rev. B.

[84] Seredin P.V., Goloshchapov D.L., Ippolitov Y.A., Vongsvivut J. (2019). A Spectroscopic Study of Changes in the Secondary Structure of Proteins of Biological Fluids of the Oral Cavity by Synchrotron Infrared Microscopy. Opt. Spectrosc..

[85] Seredin P., Goloshchapov D., Ippolitov Y., Vongsvivut J. (2020). Comparative Analysis of Dentine and Gingival Fluid Molecular Composition and Protein Conformations during Development of Dentine Caries: A Pilot Study. Vib. Spectrosc..

[86] Zhou Y., Yang Y., Liu R., Zhou Q., Lu H., Zhang W. (2023). Research Progress of Polydopamine Hydrogel in the Prevention and Treatment of Oral Diseases. Int. J. Nanomed..

[87] Zhang J., He X., Yu S., Zhu J., Wang H., Tian Z., Zhu S., Cui Z. (2021). A Novel Dental Adhesive Containing Ag/Polydopamine-Modified HA Fillers with Both Antibacterial and Mineralization Properties. J. Dent..

[88] Zhou Y.-Z., Cao Y., Liu W., Chu C.H., Li Q.-L. (2012). Polydopamine-Induced Tooth Remineralization. ACS Appl. Mater. Interfaces.

[89] Seredin P., Goloshchapov D., Kashkarov V., Ippolitov Y., Vongsvivut J. (2022). The Molecular and Mechanical Characteristics of Biomimetic Composite Dental Materials Composed of Nanocrystalline Hydroxyapatite and Light-Cured Adhesive. Biomimetics.

[90] Al-Hamdan R.S., Almutairi B., Kattan H.F., Alresayes S., Abduljabbar T., Vohra F. (2020). Assessment of Hydroxyapatite Nanospheres Incorporated Dentin Adhesive. A SEM/EDX, Micro-Raman, Microtensile and Micro-Indentation Study. Coatings.

[91] Leitune V.C.B., Collares F.M., Trommer R.M., Andrioli D.G., Bergmann C.P., Samuel S.M.W. (2013). The Addition of Nanostructured Hydroxyapatite to an Experimental Adhesive Resin. J. Dent..

[92] Goloshchapov D.L., Ippolitov Y.A., Seredin P.V. (2020). Mechanism of Interaction among Nanocrystalline Carbonate-Substituted Hydroxyapatite and Polar Amino-Acids for the Biomimetic Composite Technology: Spectroscopic and Structural Study. Results Phys..

[93] Comeau P., Willett T. (2018). Impact of Side Chain Polarity on Non-Stoichiometric Nano-Hydroxyapatite Surface Functionalization with Amino Acids. Sci. Rep..

[94] Saranya S., Samuel Justin S.J., Vijay Solomon R., Wilson P. (2018). L-Arginine Directed and Ultrasonically Aided Growth of Nanocrystalline Hydroxyapatite Particles with Tunable Morphology. Colloids Surf. Physicochem. Eng. Asp..

[95] Tavafoghi M., Cerruti M. (2016). The Role of Amino Acids in Hydroxyapatite Mineralization. J. R. Soc. Interface.

[96] Czasch P., Ilie N. (2013). In Vitro Comparison of Mechanical Properties and Degree of Cure of a Self-Adhesive and Four Novel Flowable Composites. J. Adhes. Dent..

[97] Savić D., Joković N., Topisirović L. (2008). Multivariate Statistical Methods for Discrimination of Lactobacilli Based on Their FTIR Spectra. Dairy Sci. Technol..

